# The Role of Oxidized Low-Density Lipoproteins in Atherosclerosis: The Myths and the Facts

**DOI:** 10.1155/2013/714653

**Published:** 2013-10-03

**Authors:** Giuseppe Maiolino, Giacomo Rossitto, Paola Caielli, Valeria Bisogni, Gian Paolo Rossi, Lorenzo A. Calò

**Affiliations:** Department of Medicine (DIMED), Internal Medicine 4, University of Padova, Via Giustiniani 2, 35128 Padova, Italy

## Abstract

The oxidative modification hypothesis of atherosclerosis, which assigns to oxidized low-density lipoproteins (LDLs) a crucial role in atherosclerosis initiation and progression, is still debated. This review examines the role played by oxidized LDLs in atherogenesis taking into account data derived by studies based on molecular and clinical approaches. Experimental data carried out in cellular lines and animal models of atherosclerosis support the proatherogenic role of oxidized LDLs: (a) through chemotactic and proliferating actions on monocytes/macrophages, inciting their transformation into foam cells; (b) through stimulation of smooth muscle cells (SMCs) recruitment and proliferation in the tunica intima; (c) through eliciting endothelial cells, SMCs, and macrophages apoptosis with ensuing necrotic core development. Moreover, most of the experimental data on atherosclerosis-prone animals benefiting from antioxidant treatment points towards a link between oxidative stress and atherosclerosis. The evidence coming from cohort studies demonstrating an association between oxidized LDLs and cardiovascular events, notwithstanding some discrepancies, seems to point towards a role of oxidized LDLs in atherosclerotic plaque development and destabilization. Finally, the results of randomized clinical trials employing antioxidants completed up to date, despite demonstrating no benefits in healthy populations, suggest a benefit in high-risk patients. In conclusion, available data seem to validate the oxidative modification hypothesis of atherosclerosis, although additional proofs are still needed.

## 1. Introduction

**Figure 1:**
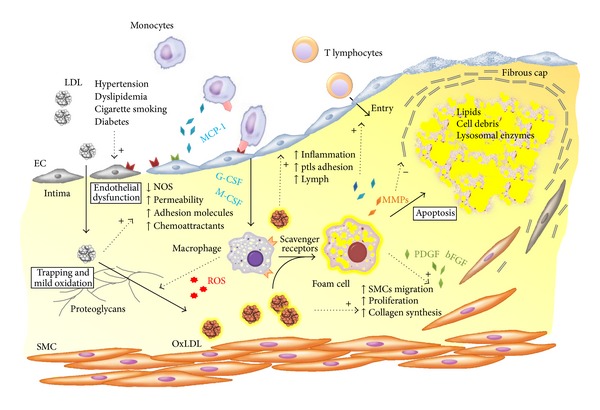
Putative pathway of oxidized low-density lipoprotein (oxLDL) in the atherogenetic process according to the oxidative hypothesis of atherosclerosis.

Recent postulates on atherosclerosis designate the appearance of qualitative changes on endothelial cells, triggered by “irritative” stimuli (e.g., hypertension, dyslipidemia, and cigarette smoking), as an early pathogenic event [1]. This process occurs at specific segments of the arterial tree, mainly branching points and bifurcations, characterized by disturbed laminar blood flow, probably owing to differences in arteries regional development [2] and to the loss of the atheroprotective effect of laminar shear stress [3]. In this setting, the endothelium expresses adhesion and chemotactic molecules and acquires an increased permeability to macromolecules, which modifies the composition of the subendothelial extracellular matrix. Hence, the entry of low-density lipoprotein (LDL) particles in the arterial wall followed by their retention through the binding of apolipoprotein B100 to proteoglycans of the extracellular matrix [4] is held to be a key-initiating factor in early atherogenesis [5]. The LDL particles trapped in the subintimal extracellular matrix are mildly oxidized by resident vascular cells [6]. They retain the capability of binding to the LDL receptor [6, 7] and to exert their proatherogenic effects [8–10], including stimulation of the resident vascular cells to produce monocyte chemotactic protein-1, granulocyte, and macrophage colony-stimulating factors. These molecules promote monocytes recruitment and their differentiation into macrophages, which are able to further promote the oxidation of LDLs [11] through myeloperoxidase and reactive oxygen species. Completely oxidized LDLs, characterized by an increased apolipoprotein B100 negative charge, are recognized by scavenger receptors on macrophages and internalized to form foam cells [12], the hallmark of the atherosclerotic lesion. Furthermore, macrophages play a key role in atherogenesis through their proinflammatory action, which involves the production of interleukin-1*β* and tumor necrosis factor (Figure 1).

Other main effectors in the development of atherosclerotic lesions are smooth muscle cells (SMCs), which are recruited from the tunica media to the subendothelial space, where they proliferate in response to mediators such as the platelet-derived growth factor. SMCs residing in the tunica intima produce extracellular matrix molecules, for example, interstitial collagen and elastin, and build the fibrous cap that overlies the growing atherosclerotic plaque. The latter entails macrophage-derived foam cells, cellular debris, and extracellular lipids, which are inefficiently cleared due to defective efferocytosis and thereby form the so-called necrotic core of the plaque [13]. 

The atherosclerotic plaque becomes clinically manifest when it reaches an advanced stage due to its blood flow-limiting effects or its destabilization with ensuing thrombosis. Unfortunately, the latter complication, which is responsible for ischemic events, is not strictly related to the degree of stenosis at angiography [14, 15] as its occurrence stands mostly on the cellular features of the plaque and particularly on the thickness of the overlying fibrous cap [16, 17]. In fact, atherosclerotic plaques prone to rupture are characterized by accumulation of inflammatory cells, mostly at the shoulder regions. These cells degrade collagen through release of collagenolytic enzymes, mainly matrix metalloproteinases (MMPs), and also reduce its synthesis by inducing SMCs apoptosis [18].

Many excellent reviews on the current knowledge of atherosclerosis are available, but few are focused on oxidized LDLs. Hence, this review examines the role played by oxidized LDLs in atherogenesis taking into account data derived by studies based on molecular and clinical approaches.

## 2. Evidence Linking Oxidized LDLs to Atherosclerosis

The oxidative modification hypothesis designates the oxidative change of LDLs as a crucial, if not mandatory, step in atherogenesis [19]. This theory originated from studies demonstrating that LDLs modified by endothelial cells, transformation entailing an oxidation process [20], could be internalized and accumulated avidly by macrophages [21, 22], leading to foam cell formation, although these cells could also be generated from macrophages internalizing native LDLs from the medium through micropinocytosis [23], or by uptake of aggregated LDLs or LDL immune complexes.

Several potential mechanisms can explain how LDL oxidative modification occurs within the arterial wall *in vivo*. A major role has been proposed for the 12/15-lipoxygenase [24, 25] because (1) it is expressed in atherosclerotic plaques but not in normal vessels [26] and (2) its inhibition was associated with decreased oxidation of LDLs [27] and reduced atherosclerosis in animal models [25, 28, 29]. Myeloperoxidase, a heme enzyme secreted by neutrophils and monocytes/macrophages, is another suggested agent. It was found in human atherosclerotic plaques [30] and modifies LDLs, thus increasing their affinity for CD36 and SR-A [31, 32], the scavenger receptors mediating the uptake of oxidized LDLs by macrophages. Nitric oxide synthase (NOS) and nicotinamide adenine dinucleotide phosphate (NADPH)-oxidase are other putative players as their products nitric oxide and superoxide anion, respectively, can combine to form the strong oxidizing agent peroxynitrite.

Native LDLs are internalized by macrophages at a pace too low to account for foam cells formation [33] owing to LDL receptor downregulation. Oxidative modification of LDLs increases their uptake by macrophages [20], via scavenger receptors. The latter are not downregulated in response to increased intracellular cholesterol, which explains why foam cells formation is held to occur with oxidized LDLs and not with native LDLs. 

Besides contributing to the formation of lipid-laden macrophages, oxidized LDLs exhibit a wide array of biological properties, which are deemed to promote atherosclerosis.

- Oxidized LDLs exert chemotactic activity for monocytes [34], stimulate their binding to endothelial cells [35] by inducing the expression of intercellular adhesion molecule-1 and vascular-cell adhesion molecule-1 [36], are mitogenic for macrophages [37], and promote their trapping in the intima, while limiting their egress from the arterial wall [38]. Hence, oxLDL is key for recruitment, activation, and proliferation of monocytes/macrophages in the arterial wall.
- Oxidized LDLs increase the expression of growth factors, such as platelet-derived growth factor (PDGF) and basic fibroblast growth factor (FGF) by endothelial cells and macrophages. The former stimulates migration of SMCs [39–41], while the latter induces SMCs proliferation [42].
- Oxidized LDLs stimulate collagen production by SMCs [43], thus contributing to the fibrous cap lining the atherosclerotic plaque and the expansion of the lesion size. However, they could also promote fibrous cap thinning by increasing secretion of matrix metalloproteinase 1 [44] and matrix metalloproteinase 9, decreasing production of the tissue inhibitor of metalloproteinase 1 [45], and inducing SMCs apoptosis [46]. Therefore, they can contribute to the occurrence of vulnerable plaques [16, 17]. Hence, taken together, this evidence involved oxidized LDLs in the progression of the atherosclerotic plaque and the development of its complications.
- Oxidized LDLs are cytotoxic to vascular cells [47, 48] and promote their apoptosis [49, 50] with ensuing release in the subendothelial space of lipids and lysosomal enzymes, enhancing the progression of the atherosclerotic plaque [47] and the production of the necrotic core.
- Oxidized LDLs stimulate platelet adhesion and aggregation, by decreasing endothelial production of nitric oxide, increasing prostacyclin production [51, 52], and stimulating the synthesis of prostaglandins and prostaglandin precursors [53]. Moreover, they decrease the secretion of the tissue-type plasminogen activator and increase that of plasminogen activator inhibitor-1 followed by a reduction of the fibrinolytic activity of endothelium [54–56]. Ultimately, they may determine vasoconstriction by inhibiting nitric oxide [57] and increasing endothelin production [58]. Taken together, these findings may explain the thrombotic complications of advanced atherosclerotic plaques.

## 3. *In Vivo* Models Supporting the Oxidized LDL Role in Atherosclerosis

Several studies were carried out* in vivo* in animal models where either a modulation of oxidative stress or manipulation of the scavenger receptor was undertaken, in order to prove the role of oxidized LDLs in the pathogenesis of atherosclerosis.

In an animal model of increased oxidative stress obtained through the overexpression of 15-lipoxygenase in the vascular wall, larger atherosclerotic lesions were found in LDL receptor-deficient mice [59]. However, a decreased atherosclerosis in cholesterol-fed rabbits and WHHL rabbits whose macrophages overexpressed human 15-lipoxygenase was also reported [60]. Animal models of reduced oxidative stress, instead, were obtained through knockout of oxidative stress-related genes or increasing the antioxidants: in three different knockout mouse models for 12/15-lipoxygenase, a decreased severity of atherosclerosis was seen [25, 61–64]. However, in apoE-deficient mice, the knocking out of the macrophage-specific 12/15-lipoxygenase increased the extension of atherosclerotic lesions [65].

The knockout in atherosclerosis-prone mice models of either SR-A or CD36 scavenger receptors, accounting for almost 90% of macrophage oxidized LDLs uptake [66], was demonstrated to be efficacious in decreasing the atherosclerotic burden [67, 68]. However, these results were not confirmed in another mice model with a CD36 and SR-A double knockout [69]. 

The results of these studies proved to be highly contradictory, due to the different animal models used, the different genetic background, and the unexpected consequences of gene deletions [70].

Finally, in spite of these conflicting data, support to the oxidative theory comes from extensive literature on the treatment of atherosclerosis-prone animals with antioxidants (reviewed by Witztum and Steinberg [71]). Most of these studies were carried out with probucol and demonstrated a protection from atherosclerotic lesions with the exception of the murine models, possibly secondary to a peculiar toxicity of this molecule in mice. In fact, in apoE-deficient mice fed with vitamin E, decreased atherosclerosis, paralleled with a decrease of aortic wall, plasma, and urinary F_2_ isoprostanes, a marker of oxidative stress, was observed [72].

## 4. Human Findings Supporting the Oxidized LDLs Role in Atherosclerosis

There is a wealth of literature on the association between oxidized LDLs and cardiovascular events. An important premise needs to be made beforehand, however, in that oxidation of LDLs induces immunogenic epitopes in their particles [73] with ensuing generation of antibodies against them (oxLDL Abs). Since these autoantibodies are detectable in the sera of the majority of patients with advanced atherosclerotic lesions [74], they can be viewed as *in vivo* markers of LDL oxidation. Oxidized LDLs and their involvement in atherosclerosis can therefore be assessed by two ways: (1) using murine monoclonal antibodies directed toward different oxidized LDLs epitopes and (2) determining the immunogenic response to oxidized LDLs. Both approaches have advantages and pitfalls, as reviewed in detail by Tsimikas [75].

Human studies on the association of oxidized LDLs with atherosclerosis or cardiovascular events have been highly conflicting (for rev. [76]). Some cross-sectional studies suggested a direct association of oxidized LDLs or oxLDL Abs with atherosclerosis in different vascular beds [74, 77, 78], whereas others found no association with coronary atherosclerotic burden in coronary artery disease patients [79–81]. Owing to these contradictory results, we focused on cohort studies, which are more solid and less prone to serendipitous findings, because of a lower chance for selection and recall bias [82].

Among the twenty-two cohort studies reporting on cardiovascular events, fourteen were positive [81, 83–94] (Table 1) and eight were negative [80, 95–101] (Table 2). Due to potential publication bias, the preponderance of positive results clearly does not provide a proof of the strength of the association [102]. However, it is important to highlight that three [95, 99, 100] out of eight negative studies were completed in healthy people. This carries a limitation in that the robustness of cohort studies depends on the assumption that the control group—in this case those exposed to low levels of oxidized LDLs—has features as close as possible to the group exposed to high levels of oxidized LDLs [103, 104]. Theoretically, this goal can be better accomplished in selected populations made of patients with similar risk profile, rather than in studies recruiting healthy persons. Among these negative studies, the first one reported on cardiovascular events in a large cohort of more than three thousand elderly patients who had 420 cardiovascular events after 5 years of followup [95]. Oxidized LDLs were predictive of cardiovascular events only if a multivariate analysis was not adjusted for the presence of metabolic syndrome. In the second study, which enrolled almost three thousand healthy subjects, *malonyldialdehyde*-LDL autoantibodies were not associated with cardiovascular events [99]. In the latter, similarly performed in a healthy population, *malonyldialdehyde*- and Cu-LDL autoantibodies and oxidized LDLs were not predictive of progression of carotid atherosclerosis [100]. 

The lack of association between oxidized LDLs and cardiovascular events, possibly due to lack of statistical power, was also reported in two small cohorts of high-risk end-stage renal disease [101] and diabetes mellitus patients [96]. 

Other negative studies enrolling patients with coronary heart disease [80, 97, 98] were either too small [80, 97] and with a number of cardiovascular events too low to allow detection of any effect of oxidized LDLs or had an endpoint not appropriate to study atherosclerosis because most of the cardiovascular events were coronary artery restenosis (75% of total events) [98]. Moreover, it is worth highlighting that the negative study published by Tsimikas et al. [80] was on the same cohort where an association between coronary artery atherosclerosis and oxidized LDLs was demonstrated [78]. 

Among the positive studies four out of fourteen were carried out in a healthy cohort [88, 90, 92, 94], thus exposing them to the same considerations expressed above. Moreover, it has to be pointed out that three of these studies were completed in the same cohort, the Brunick study, at different time points of follow up, that is, 5 [90], 10 [92], and 15 years [94], and all demonstrated a predictive value of oxidized LDLs on cardiovascular events, contradicting the results on carotid artery atherosclerosis [100] on the same population.

Other studies demonstrating an association between oxidized LDLs and cardiovascular events enrolled small cohorts of either high-risk patients [83–86] or coronary artery disease patients [87, 89, 105]. Therefore these positive results could be secondary to serendipitous findings, as suggested by the opposite results on similar cohorts of end-stage renal failure patients where high oxLDL Abs titer was associated to either low [84] or high [86] cardiovascular mortality.

Two studies reported an association of oxidized LDLs with cardiovascular events in diabetics [93] and acute coronary syndrome patients [91]. Finally, using a prospective cohort study design and an unequivocal definition of the coronary artery disease phenotype, we reported the association of oxLDL Abs with cardiovascular mortality and cardiovascular events in more than 700 coronary artery disease patients [81]. 

In conclusion, most cohort studies reported an association between oxidized LDLs and cardiovascular events or mortality, in particular those including either a very high-risk population, that is, with end-stage renal disease and diabetes, or coronary artery disease patients. However, despite being an appealing hypothesis, the oxidation theory of atherosclerosis is not conclusively corroborated by observational studies, which have conflicting results, probably owing to the enrolment selection criteria and low statistical power.

## 5. Clinical Trials on Antioxidants and the Oxidized LDL Hypothesis

The oxidative theory of atherosclerosis would be conclusively proven by the beneficial effects of oxidative stress decrease on cardiovascular events. Therefore, the analysis of controlled randomized trials on antioxidant therapy in this setting is crucial. The first report on efficacy of antioxidants on cardiovascular events in coronary artery disease patients [106] was later confirmed by further studies [107–110] (Table 3), but numerous subsequent randomized clinical trials failed to prove any benefit [111–126] (Table 4). Moreover, meta-analyses on this issue are discordant [127, 128].

An in depth analysis of these studies, however, highlighted that most of the negative studies were completed in either healthy or high-risk individuals, whereas results of clinical trials completed in patients with cardiovascular disease demonstrated the benefit conferred by antioxidants in some cases [106, 108, 109], with notable exceptions [112, 113, 117, 125]. The fact that treatment was likely given to the wrong patients, that is, with very low risk profile, can explain the failure of antioxidants trials in preventing cardiovascular events in the aforementioned negative reports [129].

Moreover, as in cohort studies, positive effects of antioxidants were witnessed in randomized trials enrolling very high-risk populations, as end-stage renal disease patients in hemodialysis, characterized by an increased oxidative stress, possibly secondary to hemolysis and hemoglobin-induced LDL oxidation [130, 131]. In these patients, with vitamin E supplementation, as tested in the SPACE trial randomizing patients to vitamin E or placebo [108], cardiovascular events were reduced by 54% and myocardial infarction by 70%. Accordingly, the potent antioxidant N-acetylcysteine showed a significant 40% decrease in the combined primary endpoint of cardiovascular events in another study [109]. After these rewarding results, we proposed the use of vitamin E coated dialysis membrane in these patients, which effectively reduces oxidative stress markers [132, 133]. Finally, in another high-risk population of diabetics carrying the haptoglobin 2-2 genotype, which is associated with inferior antioxidant protection [134], vitamin E was able to reduce the primary composite end point of cardiovascular death, nonfatal myocardial infarction, or stroke [110], even on top of statin therapy [135].

Thus, most controlled randomized trials involving the use of antioxidants provided negative results. However, administration of antioxidants to patients with known cardiovascular disease or with a very high-risk profile proved to be beneficial in a nontrivial number of studies.

## 6. Conclusions

Evidence supports on a molecular ground the oxidative hypothesis of atherosclerosis. The translation of experimental evidence in humans with studies aimed at the demonstration of the association of oxidative stress with cardiovascular events proved to be difficult and resulted in contrasting findings, particularly with administration of antioxidant therapy. However, the selection of patients either at higher risk or with cardiovascular disease provided much rewarding outcomes with numerous positive studies. It seems therefore that although this theory still needs further proofs to be definitely clarified, data available so far strengthen the pivotal role for oxidative stress in atherosclerosis.

## Figures and Tables

**Table 1:** Cohort studies demonstrating an association between oxidized low-density lipoprotein measurement and cardiovascular events.

Oxidative oxLDL test	Population under study	CV endpoints	Number of events	Followup	Findings	Reference
OxLDL Abs 4E06	326 men	IMT	na	3 years	OxLDL predicted IMT and carotid plaque progression	Wallenfeldt et al. [88]

OxLDL Abs 4E06	765 subjects	CV events	77 CV events	5 years	OxLDL predicted CV events	Tsimikas et al. [90]

OxLDL Abs 4E06	765 subjects	CV events	82 CV events	10 years	OxLDL predicted CV events	Kiechl et al. [92]

OxPL/apoB, AutoAbs MDA-/Cu-oxLDL	765 subjects	CV events	138 CV events	15 years	OxPL/apoB predicted CV events and stroke; AutoAbs predicted CV events, stroke, and ACS	Tsimikas et al. [94]

MDA-LDL	907 NIDDM	CV events, MI	152 CV events, 43 MI	3.7 years	MDA-LDL predicted MI and CV events	Lopes-Virella et al. [93]

AutoAbs Cu-oxLDL	249 ESRD	CV mortality	74 CV deaths	63 months	AutoAbs predicted CV mortality	Shoji et al. [84]

AutoAbs Cu-oxLDL	94 ESRD on hemodialysis	Total mortality	32 deaths	24 months	AutoAbs predicted mortality	Bay$\overset{´}{\text{e}}$s et al. [85]

AutoAbs Cu-oxLDL	94 ESRD on hemodialysis	CV mortality	33 CV deaths	4 years	AutoAbs predicted CV mortality	Bay$\overset{´}{\text{e}}$s et al. [86]

OxLDL Abs DLH3	246 pts with coronary angiography	CV events: cardiac death, MI, PTCA, and CABG	76 CV events	38 months	OxLDL predicted CV events	Shimada et al. [87]

AutoAbs MDA-oxLDL	734 IHD pts	CV mortality, MI, ACS, and CV events	65 CV deaths, 153 CV events	7.2 years	OxLDL predicted CV death and events	Maiolino et al. [81]

AutoAbs Cu-oxLDL	74 PTCA pts, 14 ctr	Restenosis	34 restenosis	6 months	AutoAbs predicted restenosis	Lee et al. [107]

OxLDL Abs DLH3	102 primary PTCA pts, 86 ctr	Restenosis	25 restenosis	6 months	OxLDL predicted restenosis	Naruko et al. [89]

OxLDL Abs 4E06	433 ACS pts	CV death, MI	17 CV deaths, 57 MI	2 years	OxLDL predicted MI	Johnston et al. [91]

OxLDL Abs FOH1a/DLH3	84 CHF pts (EF < 45%), 18 ctr	CV death, CV hospitalization, and fatal arrhythmia	26 CV events	780 days	OxLDL predicted CV events	Tsutsui et al. [83]

Abs: antibodies; ACS: acute coronary syndrome; AutoAbs: autoantibodies; CABG: coronary artery by-pass surgery; CHF: congestive heart failure; Crt: controls; CV: cardiovascular; ESRD: end-stage renal disease; IHD: ischemic heart disease; IMT: intima-media thickness; MI: myocardial infarction; oxLDL: oxidized low-density lipoproteins; OxPL/apoB: oxidized phospholipids on apolipoprotein B-100; PTCA: percutaneous transluminal coronary angioplasty; pts: patients.

**Table 2:** Cohort studies demonstrating no association between oxidized low-density lipoprotein measurement and cardiovascular events.

Oxidative oxLDL test	Population under study	CV endpoints	Number of events	Followup	Findings	Reference
OxLDL Abs 4E06	3033 elderly	CV events	418 IHD, 120 MI	3 years	OxLDL did not predict CV events at MV analysis	Holvoet et al. [95]

AutoAbs MDA-oxLDL	2619 subjects	IHD (angina, ACS, and IHD death); CV events (IHD + TIA/stroke)	151 IHD, 234 CV events	8 years	AutoAbs did not predict CV events	Wilson et al. [99]

OxLDL Abs 4E06, AutoAbs MDA-/Cu-oxLDL	919 subjects	Carotid atherosclerosis progression	na	5 years	AutoAbs and oxLDL did not predict CV events	Mayr et al. [100]

AutoAbs Cu-oxLDL	92 NIDDM, 80 ctr	CV events	34 CV events, 15 CV deaths	10 years	AutoAbs did not predict CV events	Uusitupa et al. [96]

OxLDL Abs 4E06	69 ESRD on hemodialysis, 33 ctr	CV events	18 CV events	43 months	OxLDL did not predict CV events	Lee et al. [101]

AutoAbs Cu-oxLDL	415 IHD	CV events	35 CV deaths/MI, 33 PTCA/CABG	5 years	AutoAbs did not predict CV events	Erkkil$\ddot{\text{a}}$ et al. [97]

OxLDL Abs 4E06	687 PTCA pts	Restenosis, CV events	135 restenosis, 181 CV events	1 year	OxLDL did not predict CV events	Braun et al. [98]

Abs: antibodies; AutoAbs: autoantibodies; CABG: coronary artery by-pass surgery; CHF: congestive heart failure; Crt: controls; CV: cardiovascular; ESRD: end-stage renal disease; IHD: ischemic heart disease; IMT: intima-media thickness; MI: myocardial infarction; NIDDM: noninsulin dependent diabetes mellitus; oxLDL: oxidized low-density lipoproteins; PTCA: percutaneous transluminal coronary angioplasty; pts: patients.

**Table 3:** Randomized controlled trials demonstrating a beneficial effect of antioxidant therapy.

Source	Patients	Inclusion criteria	Antioxidant agent	Dose	Route	Endpoints	Followup	Events
CHAOS [106]	2002	Angiographically demonstrated CAD	Vit E	400/800 IU	PO	CV death + MI; nonfatal MI	510 d	CV death: 27 vit E, 23 pl; nonfatal MI: 14 vit E, 41 pl
WHS [107]	39876	Healthy women	Vit E	600 IU q48 h	PO	Composite endpoint (CV death, MI, and stroke)	10.1 y	CV events: Vit E 482, pl 517; CV death: Vit E 106, pl 140; MI: Vit E 196, pl 195
SPACE [108]	196	Hemodialysis CV disease pts	Vit E	800 IU	PO	Composite endpoint (MI, ACS, PAD, and stroke)	519 d	Composite endpoint: Vit E 15, pl 33; CV death: vit E 9, pl 15; nonfatal MI: vit E 8, pl 18
Tepel et al. [109]	134	Hemodialysis CV disease pts	Acetylcysteine	1200 mg	PO	Composite endpoint (CV death, MI, PTCA/CABG, PAD, and stroke)	14.5 m	Composite endpoint: acetylcysteine 18, pl 33
Milman et al. [110]	1434	Diabetes mellitus Hp 2-2 genotype	Vit E	400 IU	PO	Composite endpoint (CV death, MI, and stroke)	18 m	Composite endpoint: Vit E 16, pl 33

CAD: coronary artery disease; CV: cardiovascular; d: days; DM: diabetes mellitus; HR: hazard ratio; HTN: arterial hypertension; m: months; MI: myocardial infarction; MLD: minimal luminal diameter; na: not available; PAD: peripheral artery disease, pl: placebo; PO: per os; pts: patients; RF: risk factor; vit: vitamin; y: years.

**Table 4:** Randomized controlled trials demonstrating no effect of antioxidant therapy.

Source	Number of patients	Inclusion criteria	Antioxidant agent	Dose	Route	Endpoints	Followup	Events
Virtamo et al. [111]	27271	Male smokers	Vit E, beta-carotene	50 mg, 20 mg	PO	Major coronary events (CV death, MI)	6.1 y	CV events: Vit E 519, beta-carotene 547, Vit E + beta-carotene 511, and pl 534; CV death: Vit E 212, beta-carotene 235, Vit E + beta-carotene 222, and pl 238; non-fatal MI: Vit E 307, beta-carotene 312, Vit E + beta-carotene 289, and pl 296

Rapola et al. [112]	1862	Previous MI	Vit E, beta-carotene	50 mg, 20 mg	PO	Major coronary events (CV death, MI)	5.3 y	CV events: Vit E 94, beta-carotene 113, Vit E + beta-carotene 123, and pl 94

HATS [113]	80	CAD	Vit E/C, beta-carotene, and selenium	800 IU, 1 g, 25 mg, and 100 g	PO	Composite endpoint (CV death, MI, and PTCA/CABG)	38 m	CV events: antiox 9, pl 9; CV death: antiox 0, pl 1; nonfatal MI: antiox 1, pl 4

PHS II [114]	14641	Male physicians	Vit E/C	400 IU 500 mg	PO	Composite endpoint (CV death, MI, and stroke)	8 y	CV events: Vit E 620, pl 625; Vit C 619, pl 626; CV death: Vit E 258, pl 251; Vit C 256, pl 253; MI: Vit E 240, pl 271; Vit C 260, pl 251

WACS [115]	8171	High CV risk women	Vit E/C, beta-carotene	600 IU q48 h, 500 mg, and 50 mg q48 h	PO	Composite endpoint (CV death, MI, PTCA/CABG, and stroke)	9.4 y	CV events: Vit E 708, pl 742; Vit C 731, pl 719; beta-carotene 731, pl 719; CV death: Vit E 193, pl 202; Vit C 206, pl 189; beta-carotene 211, pl 184; MI: Vit E 131, pl 143; Vit C 140, pl 134; beta-carotene 135, pl 139

PPP [116]	4495	Subjects ≥ 1 RF	Vit E	300 mg	PO	Composite endpoint (CV death, MI, and stroke)	3.6 y	CV events: Vit E 56, pl 53; CV death: Vit E 22, pl 26; MI: Vit E 22, pl 25

GISSI-prevenzione [117]	5660	Recent MI	Vit E	300 mg	PO	Composite endpoint (CV death, MI, and stroke)	3.5 y	CV events: Vit E 371, pl 414; CV death: Vit E 155, pl 193; MI: Vit E 22, pl 25

Greenberg et al. [118]	1720	Skin cancer	beta-carotene	50 mg	PO	CV death	4.3 y	CV death: beta-carotene 68, pl 59

PHS [119]	22071	Male physicians	beta-carotene	50 mg q48	PO	Malignant neoplasm; composite endpoint (CV death, MI, and stroke)	12 y	CV events: beta-carotene 967, pl 972; CV death: beta-carotene 338, pl 313; MI: beta-carotene 468, pl 489

SUVIMAX [120]	13017	Adult subjects	Vit E/C, beta-carotene, selenium, and zinc	30 mg, 120 mg, 6 mg, 100 g, and 20 mg	PO	CV ischemic events	7.5 y	CV events: antiox 134, pl 137

HPS [121]	20536	CAD, PAD, DM, and HTN	Vit E/C, beta-carotene	600 mg, 250 mg, 20 mg	PO	Composite endpoint (CV death, and MI)	5 y	CV death: antiox 878, pl 840; MI: antiox 1063, pl 1047; CV events: antiox 2306, pl 2312

HOPE [122]	9541	CV disease or DM + additional CV RF	Vit E	400 IU	PO	Composite endpoint (CV death, MI, and stroke)	7 y	CV events: Vit E 1022, pl 985; CV death: Vit E 482, pl 475; MI: Vit E 724, pl 686

Mark et al. [123]	3318	Esophageal dysplasia	Vit E/C, beta-carotene	60 IU, 180 mg, and 15 mg	PO	CV death	6 y	CV death: antiox 22, pl 35

CARET [124]	1845	Exposure to asbestos or smoke	Vit E/A	15/30 mg, 25000 IU	PO	Lung cancer incidence	5.5 y	CV death: HR 1.26 (0.99–1.61)

WAVE [125]	213	Postmenopausal women with CAD	Vit E/C	400 IU, 500 mg	PO	Change in MLD	2.8 y	CV events: antiox 10, pl 5; CV death: antiox 4, pl 2; nonfatal MI: antiox 3, pl 1

HOPE [126]	9541	CV disease or DM + additional CV RF	Vit E	400 IU	PO	Composite endpoint (CV death, MI, stroke)	4.5 y	CV events: Vit E 772, pl 739; CV death: Vit E 342, pl 328; MI: Vit E 532, pl 524

CAD: coronary artery disease; CV: cardiovascular; d: days; DM: diabetes mellitus; HR: hazard ratio; HTN: arterial hypertension; m: months; MI: myocardial infarction; MLD: minimal luminal diameter; na: not available; PAD: peripheral artery disease, pl: placebo; PO: per os; pts: patients; RF: risk factor; vit: vitamin; y: years.
